# Supplementary material for: Prognostic Genetic Biomarkers Based on Oncogenic Signaling Pathways for Outcome Prediction in Patients with Oral Cavity Squamous Cell Carcinoma
Source: Cancers (Basel). 2021 May 30;13(11):2709. doi: 10.3390/cancers13112709 (PMC8199274; doi:10.3390/cancers13112709)
Supplement: Supplementary file 1 [file cancers-13-02709-s001.zip › cancers-1217998-SI/Suppl-1217998/cancers-1217998-supplementary-2021-05-30.pdf]

cancers MDPI

Prognostic Genetic Biomarkers Based on Oncogenic Signaling Pathways for Outcome Prediction in Patients with Oral Cavity Squamous Cell Carcinoma

Wen-Lang Fan, Lan Yan Yang, Jason Chia-Hsun Hsieh, Tsung Chieh Lin, Mei-Yeh Jade Lu and Chun-Ta Liao

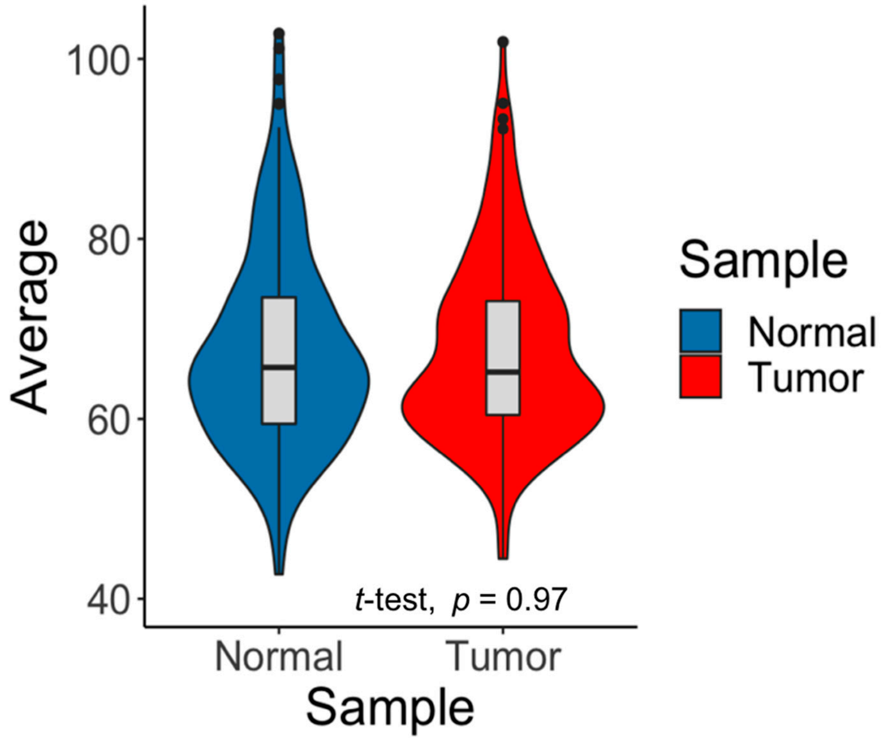

Figure S1. Violin plot showing distribution of the whole-exome sequencing depths for 165 oral cavity squamous cell carcinoma tumor sample with their matched non-tumor controls.

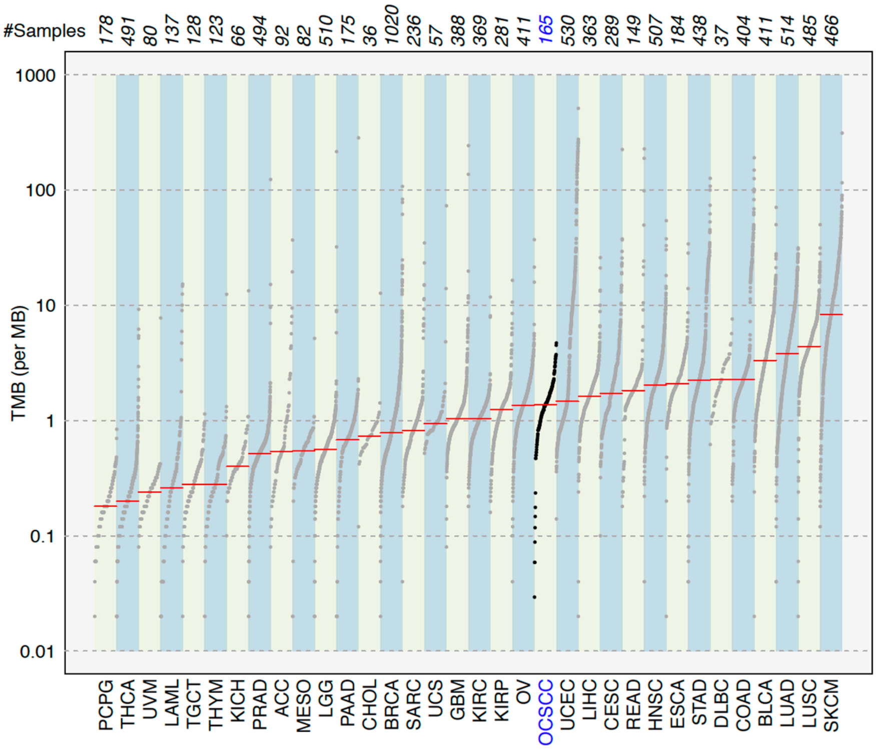

Figure S2. The landscape of tumor mutation burden (TMB) comparing cancers from various cohorts. The comparison of mutation burden of oral cavity squamous cell carcinoma (OCSCC) against 33 cohorts from The Cancer Genome Atlas (TCGA) cancer genomics dataset includes over 10,000 tumor-normal exome pairs across 33 different cancer types. The 165 OCSCC samples in this study are indicated in blue.

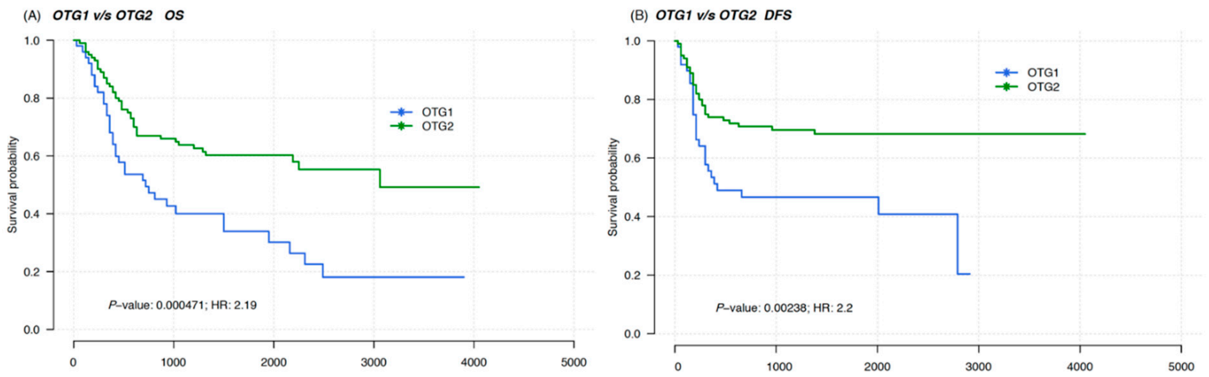

Figure S3. Kaplan–Meier curves for disease-free survival (DFS) and overall survival (OS) based on the analysis of oral cavity squamous cell carcinoma (OCSCC) tumor groups. Patients in the OTG1 group had significantly worse (**A**) OS (p = 0.000471, HR = 2.19) and (**B**) DFS (p = 0.00238, HR = 2.2) than those in the OTG2 group.

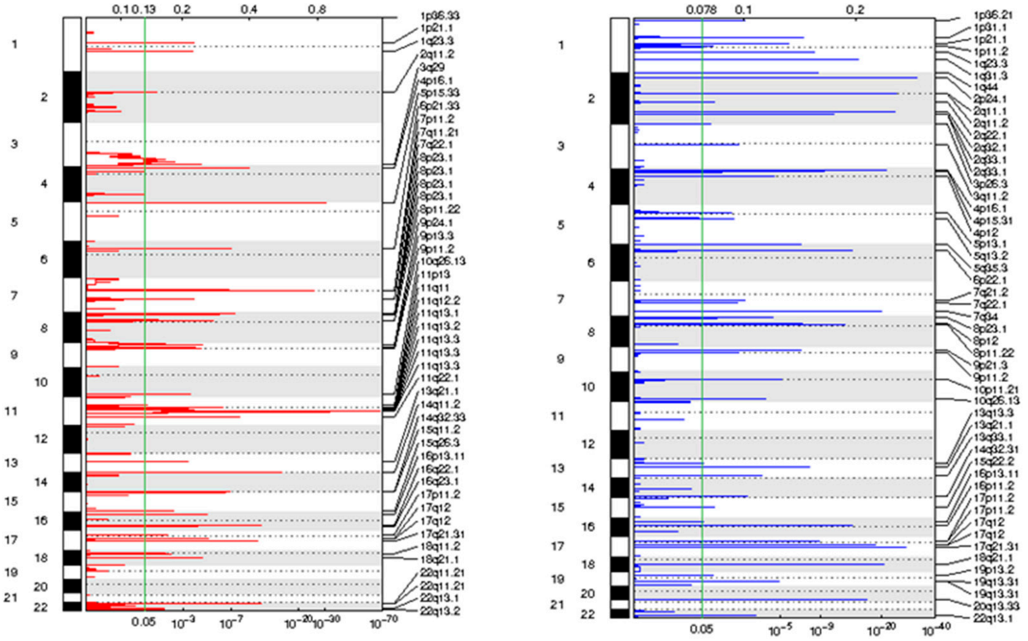

Figure S4. Plot of q-values for amplifications and deletions detected using GISTIC2. The vertical green lines denote the threshold of significance ($p < 0.05$). Deletions are indicated in blue (right panel) and amplifications in red (left panel). For deletions, two peaks were identified. The first is the most significant peak, a deletion on chromosome 9p21.3, affecting CDKN2A. The second is the proposed CDK6 deletion on chromosome 7q21.2.

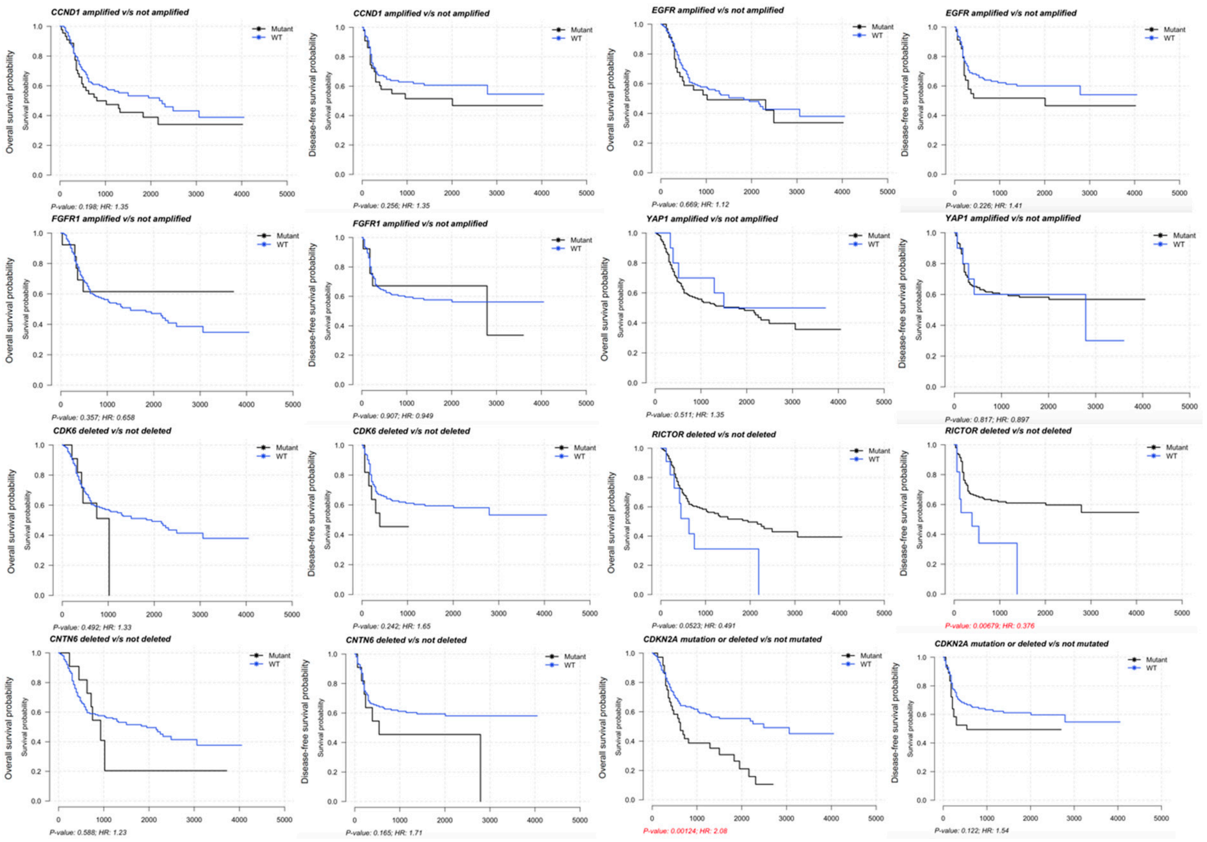

Figure S5. Kaplan–Meier curves for overall survival and disease-free survival based on the analysis of gene copy number alterations. Among the 16 mutated genes, patients with RICTOR gene deletion had significantly worse DFS (p = 0.00679), and CDKN2A gene mutations and/or deletions had significantly worse OS (p = 0.00124).

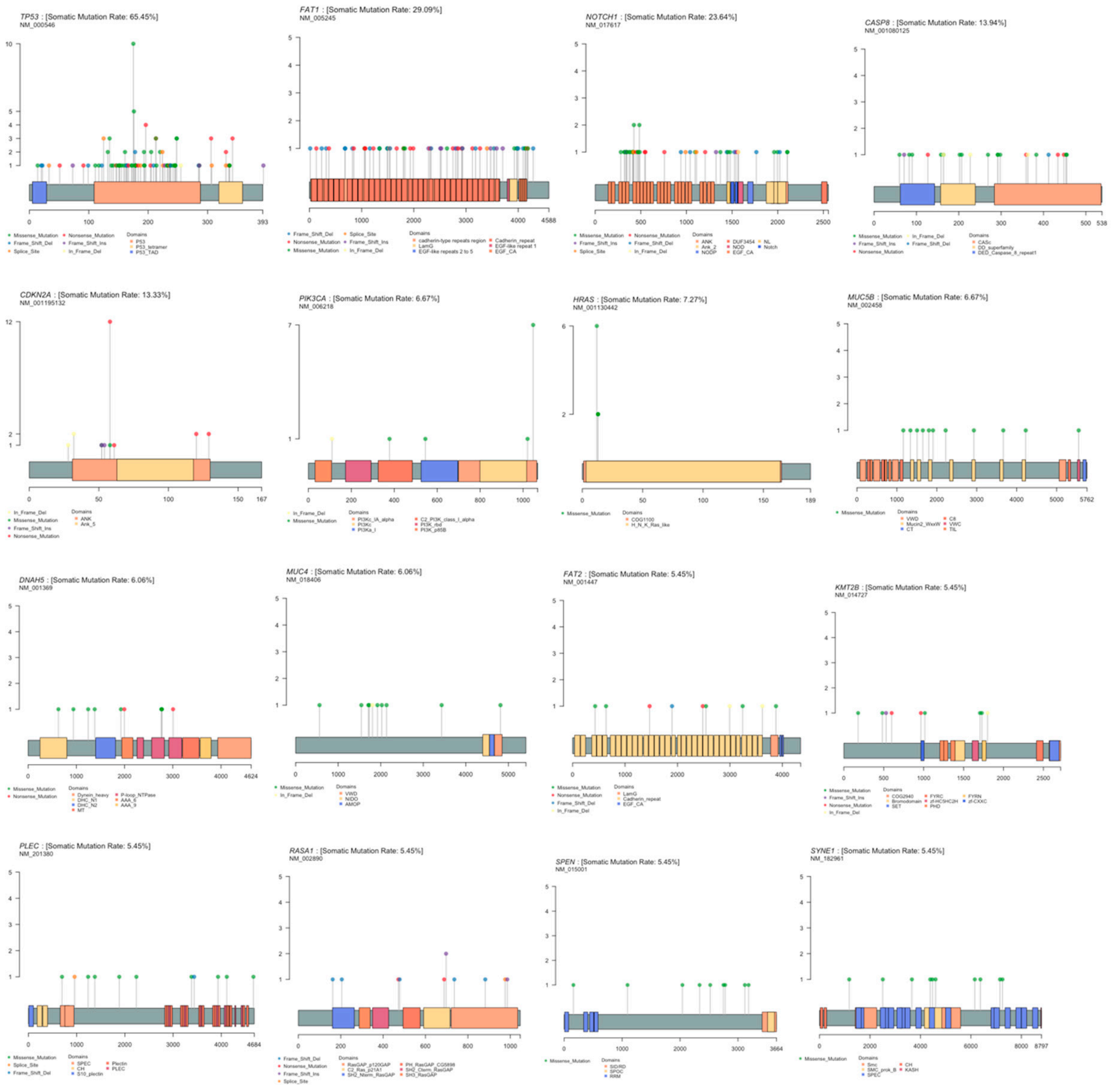

Figure S6. Lollipop plots of all mutations in the 16 significantly mutated genes. The plots were created using the maftools/cBioPortal tool in R package. Red and green lollipops denote truncations and missense mutations, respectively; violet and blue lollipops denote frameshift insertions and deletions, respectively. The height of each lollipop is proportional to the number of instances of that mutation in the dataset.

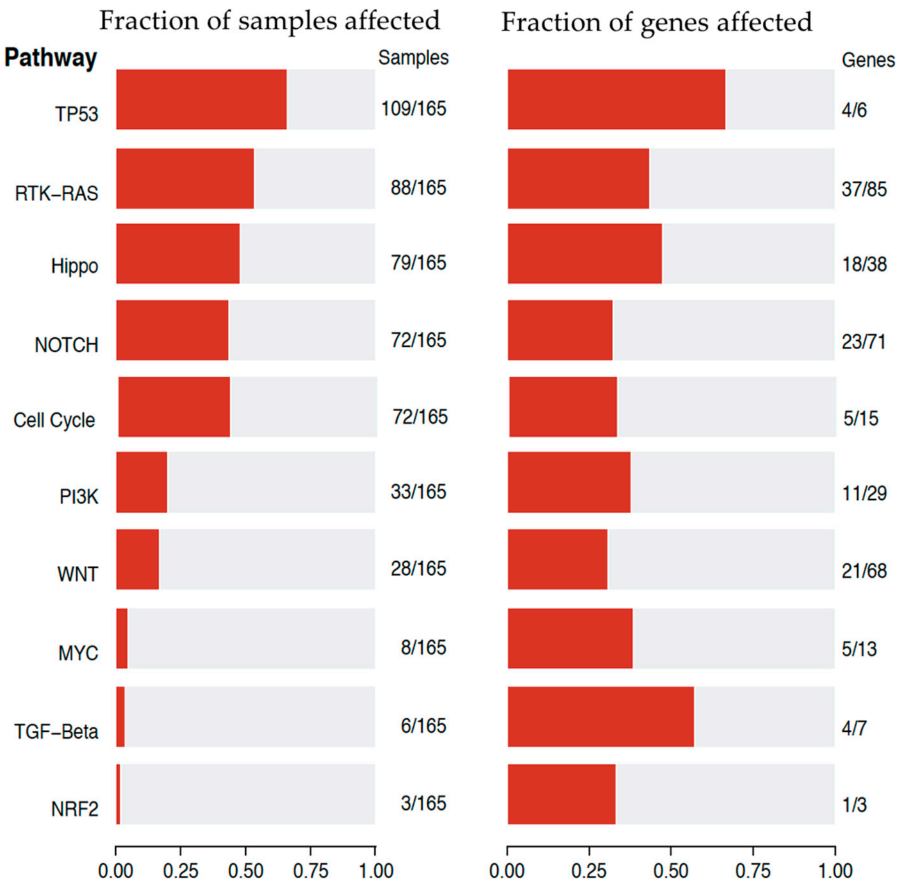

Figure S7. Mutations in oncogenic pathways of 165 oral cavity squamous cell carcinoma (OCSCC) samples. Left, fraction of samples affected in different pathways; right, fraction of genes affected in different pathways.

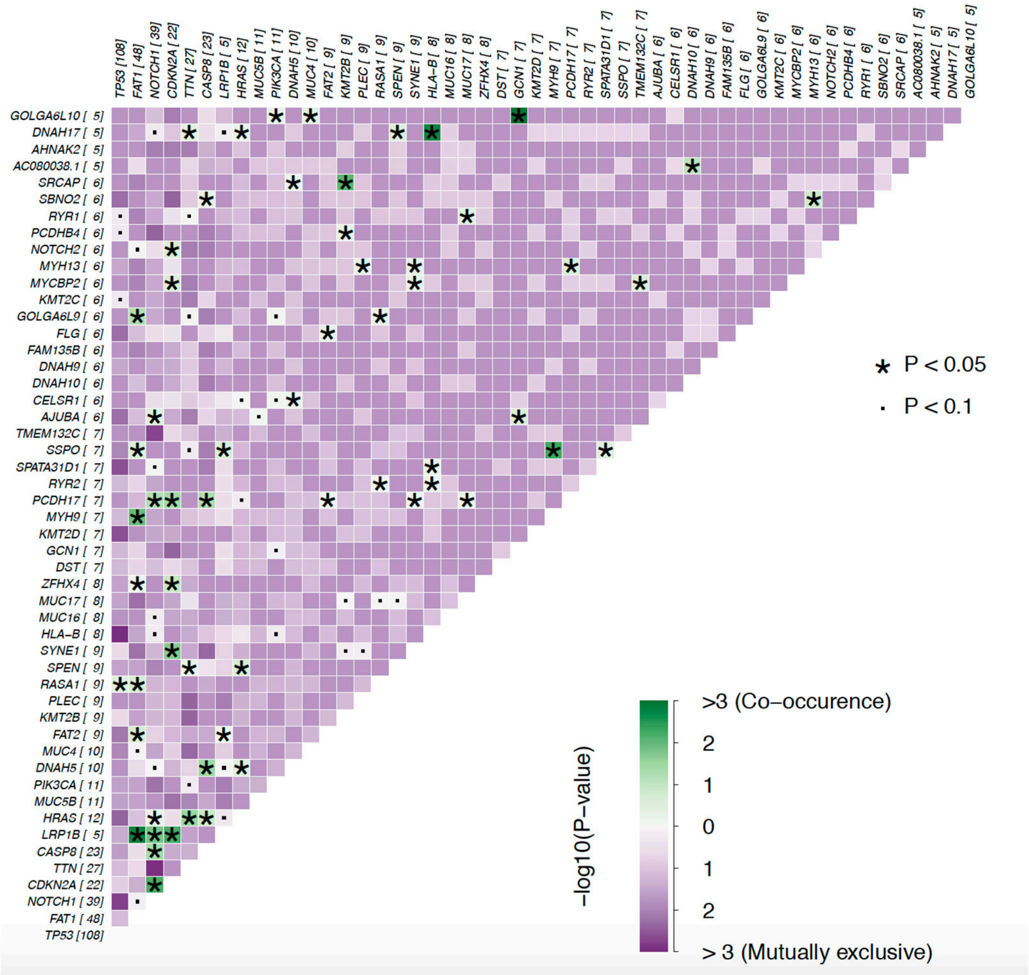

Figure S8. Mutually exclusive and co-occurring oncogene mutations in oral cavity squamous cell carcinoma. Green represents co-occurrence mutations; purple represents mutually exclusive mutations. Significant relationships are marked with asterisks ($p < 0.05$) or dots ($p < 0.1$).

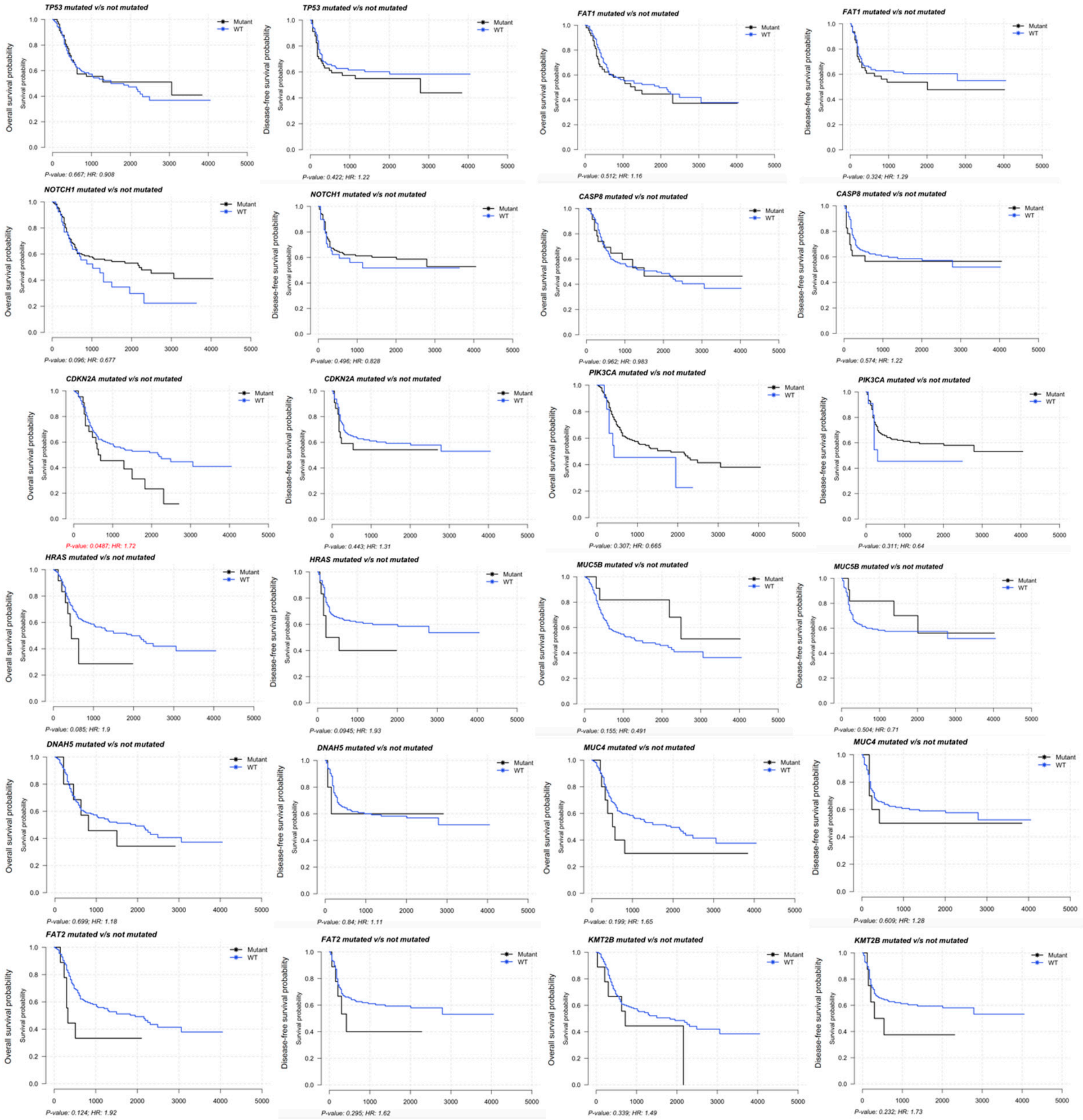
TP53 mutated v/s not mutated
P-value: 0.667; HR: 0.908
TP53 mutated v/s not mutated
P-value: 0.422; HR: 1.22
FAT1 mutated v/s not mutated
P-value: 0.512; HR: 1.16
FAT1 mutated v/s not mutated
P-value: 0.324; HR: 1.29
NOTCH1 mutated v/s not mutated
P-value: 0.096; HR: 0.677
NOTCH1 mutated v/s not mutated
P-value: 0.496; HR: 0.828
CASP8 mutated v/s not mutated
P-value: 0.962; HR: 0.983
CASP8 mutated v/s not mutated
P-value: 0.574; HR: 1.22
CDKN2A mutated v/s not mutated
P-value: 0.0487; HR: 1.72
CDKN2A mutated v/s not mutated
P-value: 0.443; HR: 1.31
PIK3CA mutated v/s not mutated
P-value: 0.307; HR: 0.665
PIK3CA mutated v/s not mutated
P-value: 0.311; HR: 0.64
HRAS mutated v/s not mutated
P-value: 0.085; HR: 1.9
HRAS mutated v/s not mutated
P-value: 0.0945; HR: 1.93
MUC5B mutated v/s not mutated
P-value: 0.155; HR: 0.491
MUC5B mutated v/s not mutated
P-value: 0.504; HR: 0.71
DNAH5 mutated v/s not mutated
P-value: 0.699; HR: 1.18
DNAH5 mutated v/s not mutated
P-value: 0.84; HR: 1.11
MUC4 mutated v/s not mutated
P-value: 0.199; HR: 1.65
MUC4 mutated v/s not mutated
P-value: 0.609; HR: 1.28
FAT2 mutated v/s not mutated
P-value: 0.124; HR: 1.92
FAT2 mutated v/s not mutated
P-value: 0.295; HR: 1.62
KMT2B mutated v/s not mutated
P-value: 0.339; HR: 1.49
KMT2B mutated v/s not mutated
P-value: 0.232; HR: 1.73
Overall survival probability
Disease-free survival probability
Survival probability
Mutant
WT

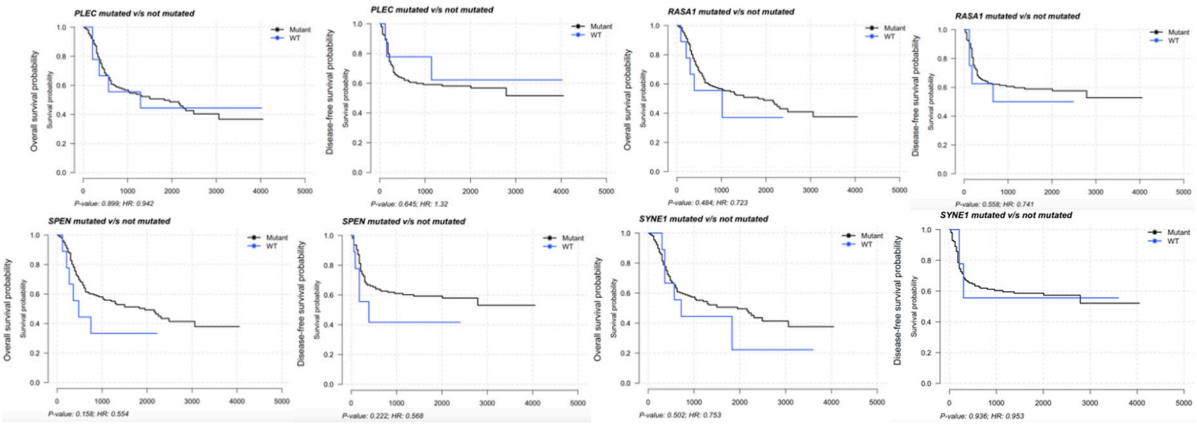

Figure S9. Kaplan-Meier survival curves of overall survival and disease-free survival. From top left to bottom right, the 8 panels show significantly mutated genes (TP53, FAT1, NOTCH1, CASP8, CDKN2A, PIK3CA, HRAS, MUC5B, DNAH5, MUC4, FAT2, KMT2B, PLEC, RASA1, SPEN, and SYNE1). Blue line, WT; black line, mutant.
